# Effect of Environmental Conditions on Copepod Size Structure and Energy Flux in the Pelagic Ecosystem of the Kuril Islands Region

**DOI:** 10.3390/biology15151293

**Published:** 2026-08-04

**Authors:** Valentina Kasyan

**Affiliations:** Laboratory of Systematics and Morphology, A.V. Zhirmunsky National Scientific Center of Marine Biology, Far Eastern Branch, Russian Academy of Sciences (NSCMB, FEB, RAS), Vladivostok 690041, Russia; valentina-k@yandex.ru

**Keywords:** copepods, size structure, NASS, NBSS, energy flux, water mass, Kuril Islands, pelagic ecosystem

## Abstract

Spatial and vertical variations in copepod abundance, carbon biomass, and size spectra are analyzed to elucidate the effects that the highly variable oceanographic conditions of the environment around the Kuril Islands exerted on the copepod size structure and energy flux in the pelagic ecosystem during the summer of 2024. Three water masses have been delineated based on hydrological characteristics: the subarctic Oyashio Current Water (OCW), the subtropical Soya Warm Current Water (SWCW), and the Kuroshio Extension Water (KEW). The total abundance of copepods decreased from OCW to KEW (OCW > SWCW > KEW), while the total carbon biomass did not differ between the OCW and SWCW but was reduced in the KEW. Clear vertical patterns of variations in average carbon biomass have been recorded: the highest peak in the SWCW, driven by medium-sized copepods above the thermocline and by large-sized copepods below it; an intermediate peak in the OCW, characterized by the dominance of large-sized copepods throughout all layers; and the lowest peak in the KEW, with small-sized copepods dominating above the thermocline and medium-sized copepods below it. Normalized abundance size spectra (NASS) and normalized carbon biomass size spectra (NBSS) were constructed for each water mass and sampling layer. The copepod productivity declined towards higher latitudes, while energy transfer efficiency and size structure stability showed a tendency to increase with lower water temperatures and with depth. The combination of chlorophyll *a* and temperature was the primary factor controlling the copepod accumulation above the thermocline, whereas the combination of dissolved oxygen and chlorophyll *a* was the primary factor below the thermocline. This study highlights the significance of each water mass to the changing pelagic ecosystem in the northwestern Pacific Ocean.

## Simple Summary

Size spectra of copepods have increasingly been used to estimate energy flux through the pelagic ecosystem. In this study, spatial and vertical variability in copepod normalized abundance size spectra (NASS) and normalized carbon biomass size spectra (NBSS) are related to oceanographic parameters of water masses in order to elucidate how environmental variability affects energy flux in the Kuril Islands region, which is an important nursery and feeding ground for pelagic fish. This study provides new data to enhance understanding of how environmental conditions influence copepod size structure, carbon biomass export, and energy transfer efficiency. The findings also underscore the role of copepods as a natural indicator of environmental health during ecosystem shifts in the northwestern Pacific Ocean.

## 1. Introduction

The Kuril Islands, located in the Northwest Pacific, stretch between the Kamchatka Peninsula in the north and Hokkaido Island in the south, separating the Sea of Okhotsk from the Pacific Ocean. Spanning several climatic zones and supporting abundant fish stocks, the region of these islands provides a valuable model for studying key physical, chemical, and biological processes. The waters along the Kuril Islands represent a dynamic system of fronts, eddies, upwelling zones, and intense mixing [1,2,3,4,5]. The hydrodynamic environment not only determines the physical conditions of the region but also influences its biological productivity, supporting the unique, biologically rich, and diverse marine pelagic ecosystem. These waters are home to abundant schools of fish (salmon, saury, herring, and walleye pollock) and numerous baleen whales (fin whales, sei whales, and humpback whales) feeding on lipid-rich zooplankton [6,7,8].

The Kuril Islands region is characterized by complex hydrodynamics resulting from a combined effect of three distinct water masses (from north to south): the subarctic Oyashio Current Water (OCW); the transitional Soya Warm Current Water (SWCW); and the oligotrophic Kuroshio Extension Water (KEW) [1,9,10,11]. The OCW, distributed along the Kuril Islands from north to southwest, warms to 10 °C in the summer and reaches a salinity of up to 33.4 psu. In contrast, the warm (up to 28 °C) and salty (up to 34.8 psu) KEW flows from the tropical Pacific Ocean into the southern part of the Kuril Islands, creating warm eddies and meanders [12,13]. The SWCW, flowing into the southern part of the Kuril Islands from the Sea of Japan through the La Perouse Strait, has a water temperature of 12–18 °C and a salinity of up to 33.9 psu in the summer [14].

Compared to traditional time-consuming approaches based on taxonomic identification, the functional approach—based on the analysis of functional characteristics (e.g., body size, trophic interactions, vertical carbon flux, remineralization rates, reproduction, life strategies)—is increasingly used to assess overall ecosystem health that closely depends on various ecological, hydrophysical, and climatic processes [15,16,17,18,19,20]. Such characteristics of pelagic ecosystems—energy transfer efficiency, productivity, and size structure stability—are particularly important, as they directly reflect the capacity of assemblages to sustain higher trophic levels and respond to environmental changes [21]. A practical way to assess these characteristics is through the analysis of size spectra, i.e., normalized abundance size spectra (NASS) and normalized biomass size spectra (NBSS), which are powerful ecological indicators providing an integrated description of community structure, energy flux, and ecosystem functioning. NASS represents the distribution of abundance across size classes and reflects the overall productivity and energy flux through the plankton community. NBSS, in contrast, represents the distribution of biomass across size classes and provides a direct measure of the standing stock of carbon in each size fraction. Together, these metrics provide a comprehensive, integrative framework for assessing ecosystem functioning and are particularly sensitive to environmental gradients, which makes them valuable tools for studying climate-driven changes in pelagic ecosystems [22].

Although the hydrography of the Kuril Islands region is relatively well documented, and the spatial patterns of zooplankton abundance and biomass distribution are reported in several studies [8,23,24,25,26,27,28], no systematic comparison of copepod size structure and energy transfer efficiency across the three major water masses (OCW, SWCW, and KEW) has been carried out using a unified size spectrum approach. The study is focused on copepods because of their high abundance in the zooplankton community, nutritional value, and significant contribution to the diets of pelagic fish. The copepod body size is closely related to metabolic rates, trophic position, and energy transfer efficiency, with shifts in size spectra being sensitive indicators of environmental changes. In particular, the vertical differences in functional features above and below the thermocline have not been examined to date. For this reason, a method with division of the vertical range of sampling into the zones above and below the thermocline is here proposed because copepod assemblages may vary significantly with depth, especially in the area where cold and warm water masses mix. The aims of this study were to elucidate how the environmental conditions associated with distinct water masses influence the copepod size structure, carbon biomass, and energy flux. The study also evaluates the usefulness of size spectra as indicators of ecosystem functioning in the Kuril Islands region. Therefore, our main hypothesis was that the spatial and vertical patterns of copepod size structure and energy flux may be closely associated with different water masses and their thermal properties. In order to test this hypothesis, spatial and vertical fluctuations in copepod abundance, carbon biomass, size spectra, and energy transfer efficiency were analyzed among the water masses along the Kuril Islands during summer, a season when pelagic fish species use this region as a feeding ground [7,29]. The present study can serve as a helpful reference for diagnosing water productivity, developing a size spectrum–water mass–productivity model, and mapping prospective or degrading feeding grounds for commercial fish species. Furthermore, it may significantly contribute to the prediction of future changes in the pelagic ecosystem under further climate-driven rearrangement of the hydrological regime in the northwestern Pacific Ocean.

## 2. Materials and Methods

### 2.1. Field Observations, Sampling, and Processing

**Figure 1:**
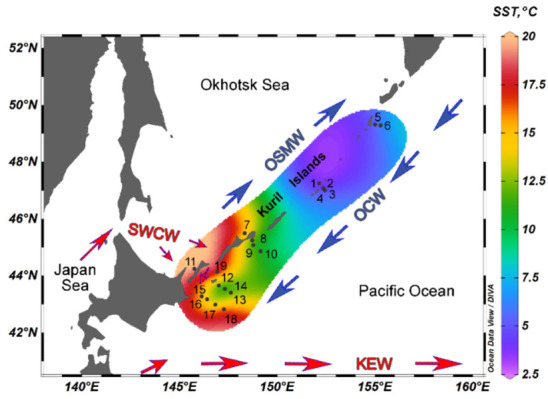
Sampling stations (1–19; black dots) and sea surface temperatures (SST, °C) around the Kuril Islands from 17 August to 10 September 2024. Acronyms are as follows: OCW, Oyashio Current Water; SWCW, Soya Warm Current Water; KEW, Kuroshio Extension Water; OSMW, Okhotsk Sea Mode Water.

Sampling was conducted onboard the R/V *Akademik Oparin* from 17 August to 10 September 2024, at 19 stations along the Kuril Islands from Onekotan Island (49° N, northern Kuril Islands) to Yuri Island (42° N, southern Kuril Islands) on both the Pacific and the Sea of Okhotsk sides (Figure 1). The environmental parameters such as water temperature (T, °C), salinity (S, psu), dissolved oxygen concentration (DO, mL L^−1^), and chlorophyll *a* concentration (Chla, µg L^−1^) in the water column were measured using a CTD profiler (SBE 19 plus V2, Sea-Bird Scientific Co., Washington, DC, USA) at each station. The depth of the thermocline was estimated for each station by determining the depth where the temperature difference between the upper and lower boundaries was more than 5 °C. The mean values of the temperature (T_up_), salinity (S_up_), dissolved oxygen concentration (DO_up_), and chlorophyll *a* concentration (Chla_up_) above the thermocline and the mean values of the same parameters (T_lo_, S_lo_, D_lo_, DO_lo_, and Chla_lo_) below the thermocline were then calculated. Copepods were collected with a WP2 net (0.25 m^2^ mouth opening, 168 μm mesh size) towed vertically in two depth zones: above the thermocline (0–50 (80) m) and below the thermocline (50 (80)–200 m). The copepod species were identified using the Marine Planktonic Copepods database [30]. Copepod abundance was expressed as the number of individuals per cubic meter (ind. m^−3^). Biomass was calculated as the product of the number of individuals and the wet weight of each individual according to Borisov et al. [31] and expressed as milligram wet weight per cubic meter (mg WW m^−3^). Wet weight was converted to carbon weight using a factor of 0.064 [32] and expressed as milligrams of carbon weight per cubic meter (mg C m^−3^). For size-specific analyses of copepods, all quantitative characteristics (e.g., abundance and carbon biomass) were grouped into three size, or body length, classes: small-sized, from 0.5 to 1 mm, including microscopic predators and detritivores, e.g., *Oithona*, nauplii and early copepodite stages; medium-sized, from 1 to 2 mm, e.g., *Pseudocalanus*, *Paracalanus*, etc.; and large-sized, >2 mm, e.g., *Calanus*, *Neocalanus*, and *Eucalanus*. The ratio of the carbon biomass of large-sized copepods to that of small-sized copepods was used as an indicator of size structure stability and energy transfer efficiency.

The water masses were classified on the basis of temperature and salinity conditions: OCW was characterized by temperatures of <5 °C and salinities of <33.0 psu; SWCW was characterized by temperatures of 5–15 °C and salinities of 33.0–33.8 psu; KEW was characterized by temperatures of >15 °C and salinities of >34.0 psu [1,10,11]. The stations were clustered into areas that corresponded to the major water masses, i.e., OCW, SWCW, and KEW. KEW was located in the Pacific waters off Yuri Island (stns. 17 and 18); SWCW was located in the Sea of Okhotsk waters from Kunashir Island to Iturup Island and from Shikotan Island to Yuri Island in the Pacific waters (stns. 7, 11, 12, 14–16 and 19); OCW was located in the Pacific waters from Onekotan Island (north) to Shikotan Island (south) (stns. 1–6, 8–10, and 13) (Figure 1).

### 2.2. Data Analysis

Cluster analyses were carried out to assess similarity between the stations. The faunal resemblances between each pair of stations and clusters were measured by the quantitative Bray–Curtis similarity index. Copepod carbon biomasses and environmental variables were square-root-transformed before analysis to obtain a normal data distribution. Analysis of similarities (ANOSIM) was used to test for differences in copepod carbon biomass data between clusters. Species diversity was estimated using the Shannon–Wiener index (H′), and species richness was estimated using Margalef’s index (D′). Pielou’s evenness index (J′) was calculated to assess the evenness of copepod species distribution across stations. To identify indicator species characterizing the currents, the Indicator Value Index (IndVaI) was used. Indicator value of the species (IndVaIi_j_) was calculated using the following formula: IndVaIi_j_ = Ai_j_ × Bi_j_ × 100%, where IndVaIi_j_ is the indicator value of the *i*th species for the *j*th group; Ai_j_ is the average abundance of the *i*th species for the *j*th group divided by the sum of the average numbers of *i*th species in all groups (shows whether the species is „concentrated” in this group); Bi_j_ is the number of samples in the *j*th group where the *i*th species occurs divided by the total number of samples in the *j*th group (shows how constant the species is within this group). An IndVaI value of >70% means that it is a strong and reliable indicator species. Major copepod species were chosen on the basis of their frequency of occurrence (i.e., occurrence at 70% of stations).

To study the size structure of copepods, their length-specific distributions between different water masses and sampling layers were plotted as log_2_-transformed average abundance against log_2_-transformed total length. This methodology can be used to generate normalized abundance size spectra (NASS) and normalized carbon biomass size spectra (NBSS) according to the equation of Sabetta et al. [33]. NASS and NBSS were calculated for each sample as log_2_(A_i_ or B_i_/ΔS) = *b* + *a* × log_2_(S_i_), where A_i_ is the copepod abundance (A, ind. m^−3^) or carbon biomass (B, mg C m^−3^), log_2_(S_i_) is the size class, ΔS is the size interval (mm), *a* is the slope, and *b* is the intercept. The slope (*a*) represents energy transfer efficiency, the intercept (*b*) indicates copepod productivity, and the regression coefficient (R^2^) is a proxy to estimate copepod assemblage stability [22]. In the case of a strongly negative (steep) regression slope of NASS or NBSS, the abundance or carbon biomass declines rapidly with increasing body size, which reflects an assemblage dominated by small-sized individuals. A weakly negative or near-zero regression (flatter) slope indicates that the abundance or carbon biomass is more evenly distributed across the size classes, with a substantial contribution from large-sized individuals. Linear NASS and NBSS are crucial metrics for characterizing ecosystem energy transfer efficiency and productivity [34,35]. The Mann–Whitney *U*-test was used to compare NASS and NBSS slopes, intercepts, and size diversities between the three water masses under study. During the NASS and NBSS analysis, smaller copepods may have been underestimated due to the mesh size. This did not affect our main conclusions, as the observed patterns were consistent across water masses and sampling layers.

Principal component analysis (PCA) was based on a correlation matrix to represent associations between environmental variables (mean water temperature, salinity, dissolved oxygen and chlorophyll *a* concentrations) above and below the thermocline. All environmental variables were log_10_(*x* + 1)-transformed and normalized prior to analysis. The effects of the environmental variables on the copepod size structure were analyzed by redundancy analysis (RDA). Multicollinearity among environmental variables was assessed using variance inflation factors (VIFs) prior to the RDA. VIFs were <5, and, therefore, all predictors were retained in the RDA. This analysis was based on the carbon biomass of copepods from three size classes and the environmental variables in the two sampling layers. Biotic data were log_10_(*x* + 1)-transformed and environmental variables (T, S, DO, and Chla) were standardized to zero mean and unit variance. PCA and RDA were selected as the primary analytical approaches because they provide complementary insights: PCA summarizes overall patterns in data, while RDA quantifies the relative influence of environmental drivers. In addition, Spearman’s rank-order correlation analysis was performed to clarify relationships between various functional characteristics of copepod assemblages and environmental variables.

One-way ANOVA (based on the normality of the environmental variable), followed by Tukey’s post hoc test, was carried out to evaluate differences in carbon biomasses and environmental variables among the copepod assemblages identified in the cluster analysis. All statistical analyses and calculations were performed using Primer 6.0 [36], R package [37], and PAST 4.05 [38]. Data, including water temperature, abundance, carbon biomass, diversity indices, and other variables, were visualized using Ocean Data View and ChiPlot (https://www.chiplot.online/) (accessed on 7 December 2025).

## 3. Results

### 3.1. Hydrography, Copepod Abundance, Carbon Biomass, and Diversity

During the summer season, the temperature and salinity above the thermocline ranged from 2.9 to 19.2 °C and from 32.9 to 34.2 psu, respectively. Below the thermocline, the temperature and salinity values were the lowest, within 1.3–5.9 °C and 32.8–33.5 psu, respectively. Both temperature and salinity showed a tendency to increase from north to south along the Kuril Islands (Table S1). The highest chlorophyll *a* concentrations were recorded from the Pacific waters off Onekotan; the highest oxygen concentrations, suggesting local mixing, were from the Pacific waters off the southern Kuril Islands.

**Figure 2:**
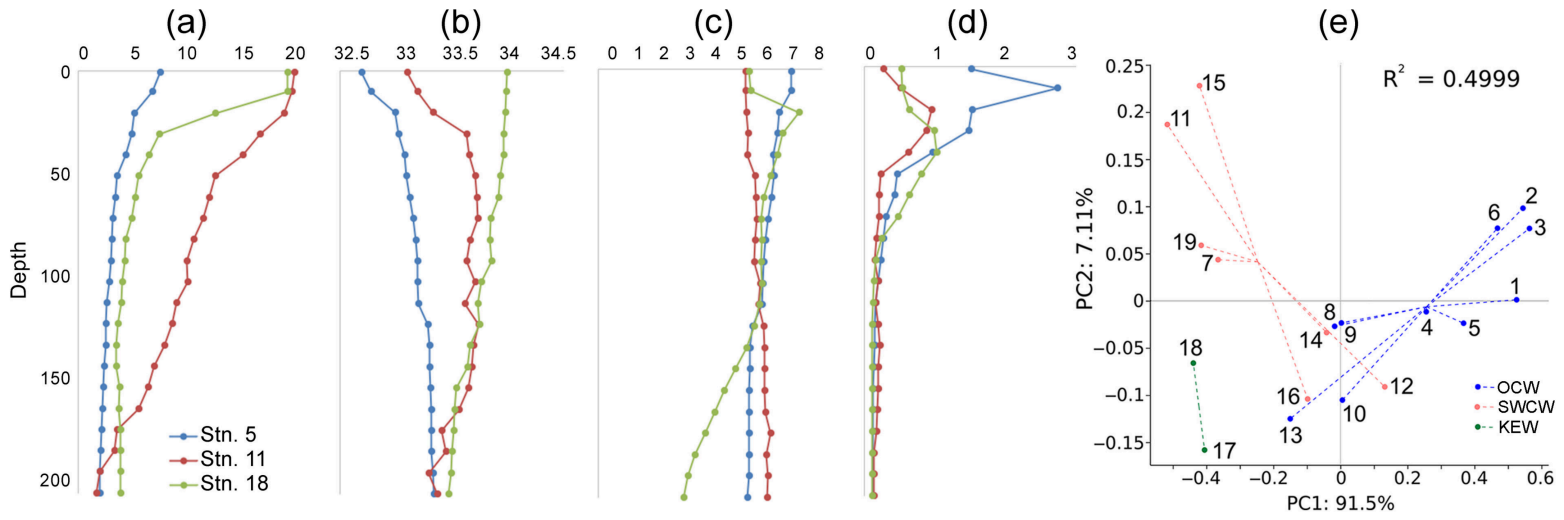
Vertical profiles of (**a**) temperature (°C), (**b**) salinity (psu), (**c**) dissolved oxygen concentration (mL L^−1^), and (**d**) chlorophyll *a* concentration (μg L^−1^) in different water masses. (**e**) Principal component analysis (PCA) of sampling stations on the basis of environmental variables around the Kuril Islands in summer. Station 5 is located in the Oyashio Current Water (OCW), station 11 is located in the Soya Warm Current Water (SWCW), and station 18 is located in the Kuroshio Extension Water (KEW). The numerals indicate sampling stations.

The water masses along the Kuril Islands were characterized by distinct flow patterns. The subarctic OCW was colder (2.3–8.0 °C), less salty (32.8–33.2 psu), had a higher Chla content (>1.0 µg L^−1^) (Table S1), and extended along the Pacific coast of the Kuril Islands. In contrast, the subtropical SWCW and KEW were the warmest (with temperatures of up to 16.8 and 17.6 °C, respectively), relatively more salty (33.2–33.9 and 33.7–34.2 psu, respectively), with a high Chla content (>6.0 mL L^−1^) (Table S1), and were located off the southern Kuril Islands. The deep Chla maximum was at a depth of about 5–10 m in the OCW, which corresponded to lower temperature and salinity levels (Figure 2a,b). The SWCW and KEW showed a very small vertical gradient of Chla with a peak at about 30–40 m (Figure 2c,d). The PCA indicated a distinct difference in the water masses, albeit with a more pronounced overlap between the OCW and SWCW than between the KEW and OCW and between the KEW and SWCW (Figure 2e). The OCW and SWCW exhibited a distinct vertical gradient. At stns. 12, 14, and 16, the SWCW was present above the thermocline; the OCW, below the thermocline. At the rest of the stations, the SWCW was present both above and below the thermocline.

**Figure 3:**
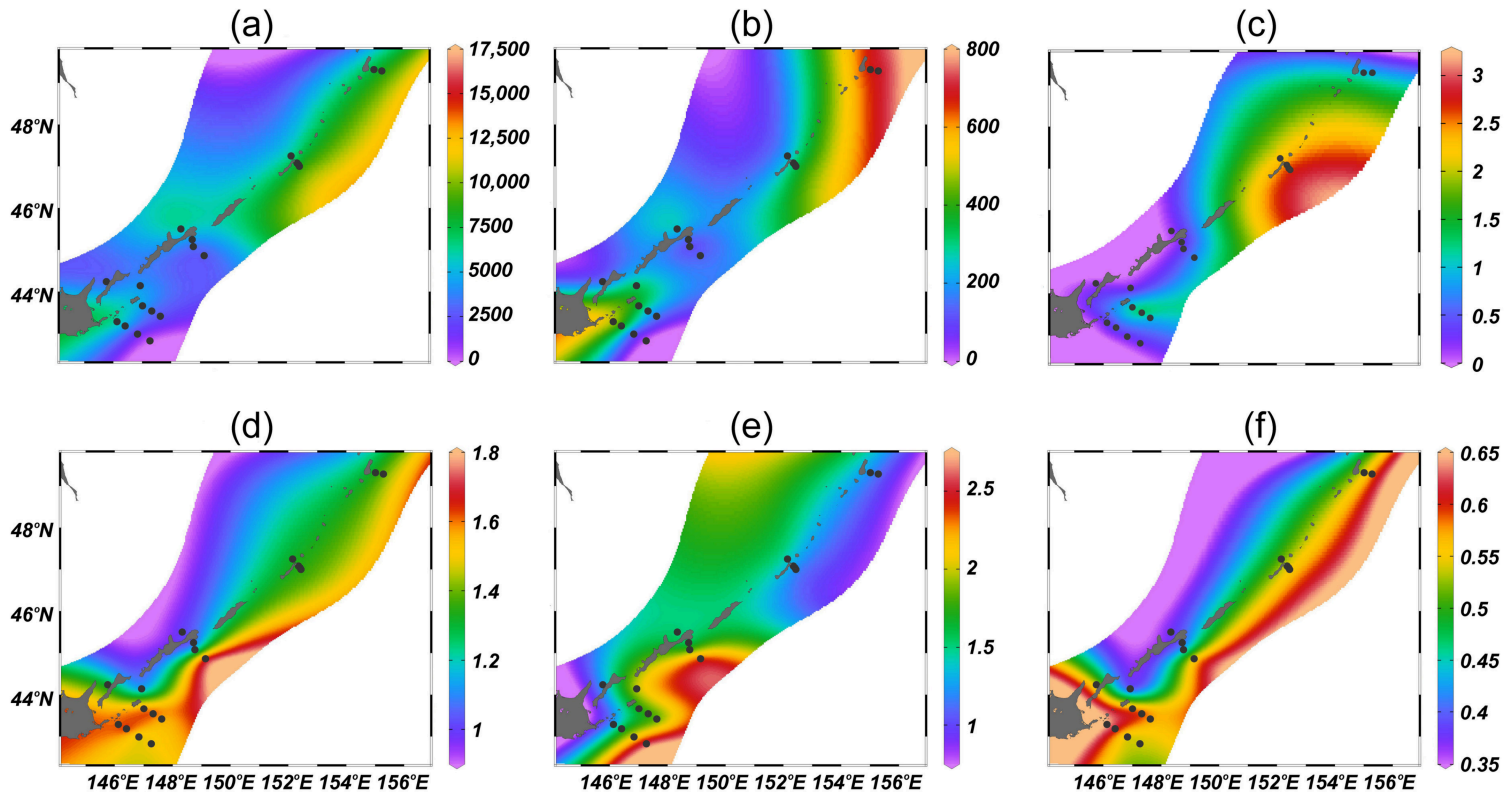
Copepod variables (integrated for the layer from 0 m to the bottom) in the waters around the Kuril Islands during the summer of 2024: (**a**) abundance (ind. m^−3^); (**b**) carbon biomass (mg C m^−3^); (**c**) ratio of carbon biomass of large-sized copepods to that of small-sized copepods. Copepod diversity indexes: (**d**) Shannon–Wiener’s index; (**e**) Margalef’s index; (**f**) Pielou’s evenness. The black dots indicate sampling stations.

The total abundance and carbon biomass of copepods varied over wide ranges (Table S1). The maximum abundances were recorded in the northern and central parts of the study area; the highest carbon biomass values were from the northernmost stations, showing higher phytoplankton concentrations (Figure 3a,b). Phytoplankton cells were not counted, and phytoplankton concentration was estimated visually in the samples. On the basis of abundance, the waters could be arranged in the following order: OCW > SWCW > KEW. The carbon biomass values did not differ between the OCW and SWCW but were reduced in the KEW and were the highest in the Pacific waters than in the Sea of Okhotsk waters. The ratio of the carbon biomass of large-sized copepods to that of small-sized copepods was higher in the northern part of the study area, especially in the Pacific waters off Simushir Island (Figure 3c). The diversity of copepods (Shannon–Wiener’s index) varied from 0.9 to 1.7 and tended to be higher in the southern part of the study area (Figure 3d). Margalef’s index demonstrated the same distribution as the Shannon–Wiener index (Figure 3e). Pielou’s evenness values were higher in the Pacific waters along the Kuril Islands, which showed a latitudinal gradient in the copepod structure (Figure 3f).

The carbon biomass of copepods displayed significant vertical variations. Above the thermocline, maximum mean carbon biomass values were recorded from the stations influenced by the SWCW, with an integrated mean temperature of 14.6 °C, salinity of 33.5 psu, chlorophyll *a* concentration of 0.98 µg L^−1^, and oxygen concentration of 5.9 mL L^−1^. Median estimated carbon biomass values were in the OCW with an integrated mean temperature of 7.6 °C, salinity of 33.0 psu, chlorophyll *a* concentration of 0.8 µg L^−1^, and oxygen concentration of 6.2 mL L^−1^. The lowest values were in the KEW with an integrated mean temperature of 17.7 °C, salinity of 33.9 psu, chlorophyll *a* concentration of 1.0 µg L^−1^, and oxygen concentration of 6.5 mL L^−1^. Below the thermocline, the copepod carbon biomass showed a tendency to increase from the southern part of the study area located within the KEW and SWCW to the northern part in the OCW, with a decrease in temperature, salinity, and oxygen values and an increase in chlorophyll *a* concentration (Table 1).

According to the IndVaI index, certain indicator species were associated with only one of the water masses. OCW was indicated by five species: *Neocalanus plumchrus* and *Pseudocalanus newmani*—significantly associated with the layer above the thermocline (*p* < 0.05, ANOVA)—and *Eucalanus bungii*, *Neocalanus cristatus*, and *Pseudocalanus minutus*—significantly associated with the layer below the thermocline (Table 2). SWCW was indicated by four species: *Clausocalanus arcuicornis*, *Paracalanus parvus*, and *Mesocalanus tenuicornis* were significantly associated with the layer above the thermocline, while *Metridia pacifica* was significantly associated with the layer below the thermocline. KEW was indicated by two significant taxa: *Oithona similis* and copepod nauplii, showing high values of indication. Additionally, *Calanus pacificus* and *P. minutus* were indicative of Pacific waters, while *P. newmani* was indicative of Sea of Okhotsk waters. The rest of the species were not observed as indicators of any of the water masses, which suggested their wide ecological valence and uniform distribution.

### 3.2. Copepod Assemblages, Size Spectra, and Energy Transfer Efficiency

**Figure 4:**
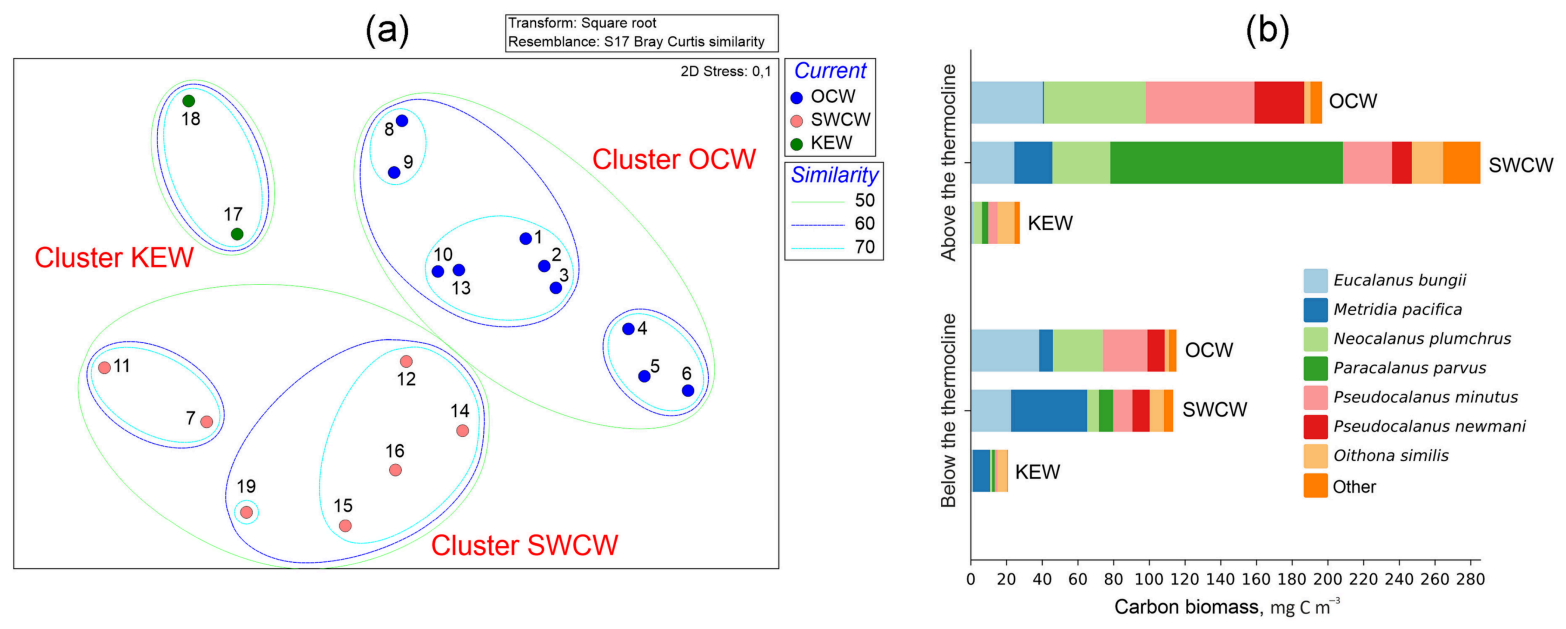
(**a**) Non-metric multidimensional scaling ordination plot of stations based on square-root-transformed copepod carbon biomass (the numerals indicate sampling stations). (**b**) Vertical distributions of average carbon biomass over major species in identified clusters.

The results of the non-metric Multidimensional Scaling (nMDS) analysis showed that the copepod size structure varied across the three water mass areas (Figure 4a). The cluster of OCW stations was characterized by the dominance of species from the large-sized class (with a copepod body length >2 mm) and was located in the northern and central part of the Kuril Islands region influenced by the Oyashio Current Water. The cluster of SWCW stations, characterized by the highest carbon biomass and the dominance of the medium-sized class (with a copepod body length from 1 to 2 mm), occurred in the southern areas influenced by the Soya Warm Current Water. In contrast, the cluster of KEW stations, characterized by the lowest carbon biomass and dominated by the small-sized class (with a copepod body length from 0.5 to 1 mm), was found at the southernmost stations influenced by the Kuroshio Extension Water (Table 3). ANOSIM showed that the copepod community structure differed more significantly between the water masses (OCW, SWCW, KEW; R = 0.717, *p* < 0.001) than between the Pacific and Sea of Okhotsk areas (R = 0.319, *p* < 0.001).

In terms of size class composition, the large-sized copepods *Eucalanus bungii*, *Neocalanus plumchrus*, the medium-sized copepods *Metridia pacifica*, *Paracalanus parvus*, *Pseudocalanus minutus*, *Pseudocalanus newmani*, and the small-sized copepods *Oithona similis* were dominant species at the stations. Among the seven major copepod species, *N. plumchrus* was predominantly distributed in each study layer, showing a higher carbon biomass in the OCW than in the SWCW and KEW clusters (Figure 4b). In contrast, *P. parvus* and *M. pacifica* were mainly observed above and below the thermocline, respectively, with a higher carbon biomass in the SWCW clusters. *Pseudocalanus minutus* and *E. bungii* were primarily found above and below the thermocline, respectively, and also exhibited a higher carbon biomass in the OCW cluster (Figure 4b). All three regional patterns of variation in average carbon biomass were also recorded. The first pattern had an abrupt peak in the SWCW, which was formed by the copepod *P. parvus* above the thermocline and by the copepods *E. bungii* and *M. pacifica* below the thermocline. The second pattern was observed at the stations located within the OCW and was characterized by the dominance of *E. bungii*, *N. plumchrus*, and *P. minutus* in all study layers. The third pattern, observed in the KEW, was characterized by the dominance of *O. similis* above the thermocline and *M. pacifica* below the thermocline.

Vertical distributions of abundance and carbon biomass of the copepod size classes between the water masses are shown in Table 3. In all the study layers, the proportions of total carbon biomass and total abundance of the large-sized class in the KEW and SWCW were lower than in the OCW. In contrast, the proportions of total carbon biomass and total abundance of the small-sized class in the OCW and SWCW were lower than in the KEW. In terms of total carbon biomass, large-sized individuals above the thermocline accounted for the largest proportion in the OCW, while medium-sized and small-sized individuals were most abundant in the SWCW and KEW, respectively. Below the thermocline, the relative proportions of the large-size class were greater in the OCW than in the SWCW and KEW. In contrast, the proportions of the small-sized class were greater in the KEW than in the SWCW and OCW (Table 3). The distribution of abundance across the three copepod size classes above the thermocline was comparable to the carbon biomass distribution. Below the thermocline, the highest proportions of large-sized copepods were found in the OCW and SWCW; medium-sized copepods, in the KEW and SWCW; small-sized copepods, in the OCW.

**Figure 5:**
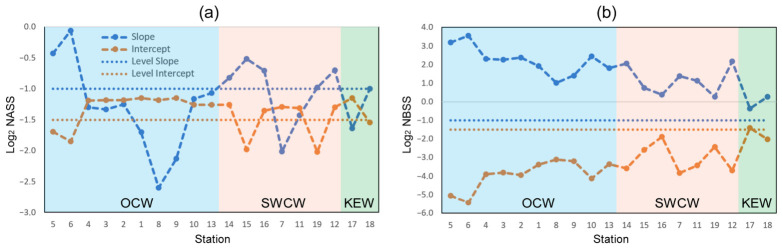
Spatial variations in (**a**) normalized abundance size spectra (NASS) and (**b**) carbon biomass size spectra (NBSS) at stations of OCW (blue area), SWCW (peach area), and KEW (green area).

The NASS slope ranged from −0.06 to −2.6 (Figure 5a). The steepest slope was observed at stn. 8 off Iturup Island, while the flattest slope was at stn. 6 off Onekotan Island. In the areas north (stns. 5 and 6) and south of the Kuril Islands (stns. 12, 14–16 and 19), the NASS slope was flatter, thus indicating a uniform distribution of abundances across the size classes. In the other areas, especially at the stations off Iturup Island, the NASS values formed a steeper slope, which indicated a numerical dominance of small-sized copepods and an extremely rapid decrease in their abundance with increasing body size. The overall mean NASS slope along the Kuril Islands showed a negative relationship between the copepod body size intervals and the abundance, with the abundance decreasing towards the larger size classes. The NBSS slopes of all sampling stations had positive values (which was a noteworthy phenomenon), except for the slope at stn. 17, which showed a negative value, thus indicating a normal model of energy transfer efficiency (Figure 5b). The NBSS intercept ranged from −1.42 to −5.45. The NBSS intercept was higher south (mean ± SD: −2.7 ± 0.4) than north of the Kuril Islands (mean ± SD: −3.9 ± 0.7).

The parameters (slope, intercept, and coefficient of determination R^2^) of NASS and NBSS were estimated for each water mass (Table 4). The NASS analysis demonstrated a significantly steeper slope and intercept in the areas of the OCW and KEW (Mann−Whitney *U* test, *p* < 0.00005), which was associated with the dominance of small-sized copepods, whereas the community stability was higher in the OCW than in the KEW. The slope and intercept in the SWCW were less steep, indicating an increase in the proportion of large-sized copepods in the total population and a less stable community. The NBSS analysis showed significant positive slopes in all the study areas except for KEW, which was associated with the dominance of large-sized copepods in the total carbon biomass (mean slope 1.59 ± 0.31; Mann−Whitney *U* test, *p* < 0.01). On the basis of NBSS slope (energy transfer efficiency), the waters could be ranked in the following order: KEW < SWCW < OCW. On the basis of NBSS intercept (productivity), KEW > SWCW > OCW. On the basis of predominance of large-sized class, KEW < SWCW < OCW. On the basis of NBSS R^2^ (size structure stability), KEW < SWCW < OCW (Table 4).

**Figure 6:**
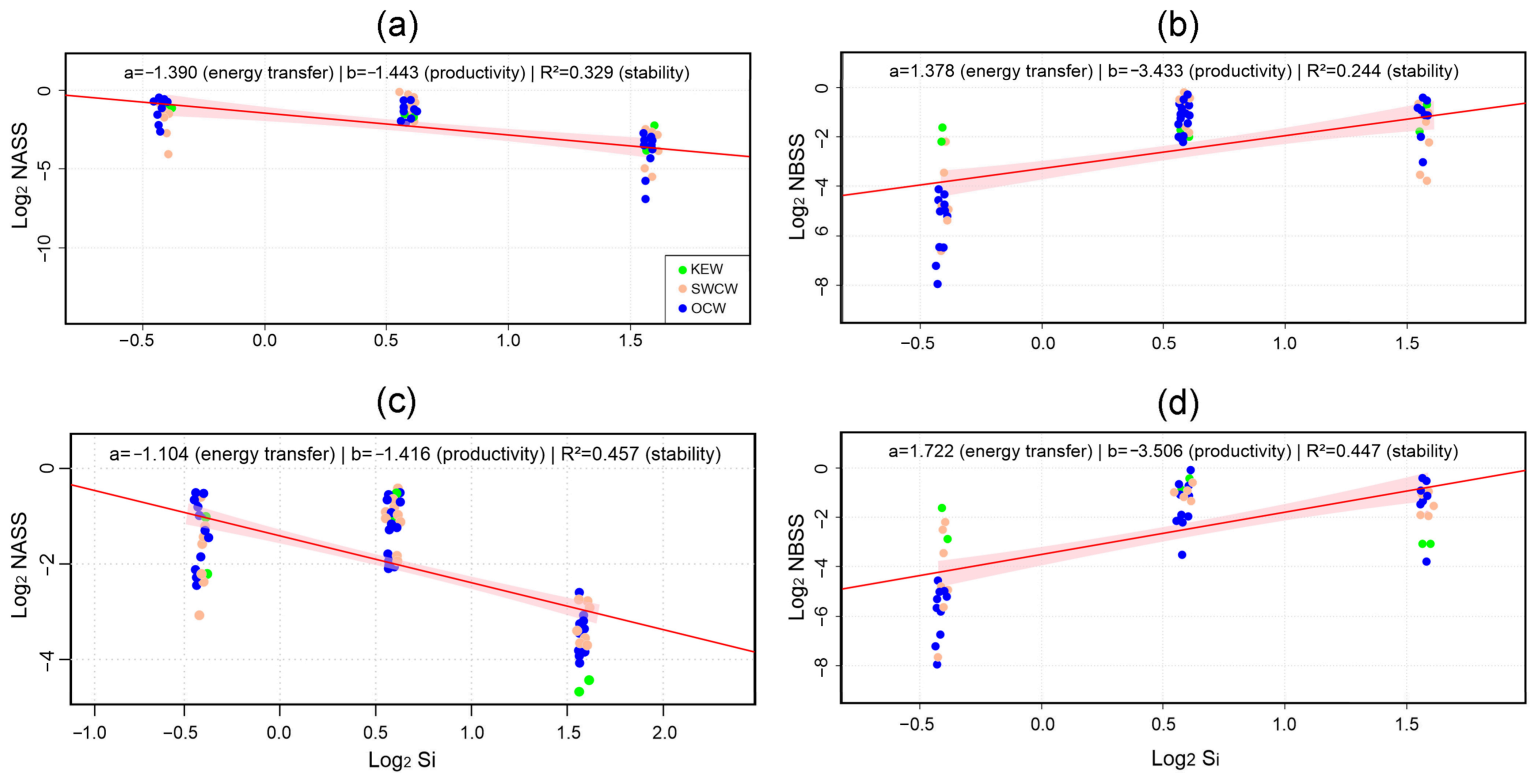
(**a**,**c**) Normalized abundance size spectra and (**b**,**d**) carbon biomass size spectra (**a**,**b**) above the thermocline and (**c**,**d**) below the thermocline off the Kuril Islands. The blue, peach, and green dots represent stations in OCW, SWCW, and KEW, respectively. The shaded areas indicate the 95% confidence interval.

The parameters of NASS and NBSS were constructed for each sampled layer (Figure 6). Above the thermocline, the NASS slope was the steepest and was characterized by the dominance of the large-, medium-, and small-sized classes with equivalent proportions. Below the thermocline, the NASS slope was the flattest, with the dominance of small-sized (43%) and medium-sized (47%) classes of copepods. With equal values of NASS intercept between the study layers, the NASS R^2^ values were higher below than above the thermocline (Figure 6a,c). The NBSS slopes for each layer had positive values, especially below the thermocline, which could be explained by the dominance of large- and medium-sized classes (in total making up 93%). The NBSS R^2^ was higher below the thermocline, and the NBSS intercepts were low for both study layers (Figure 6b,d).

### 3.3. Relationships Between Copepod Size Structure and Environmental Variables

**Figure 7:**
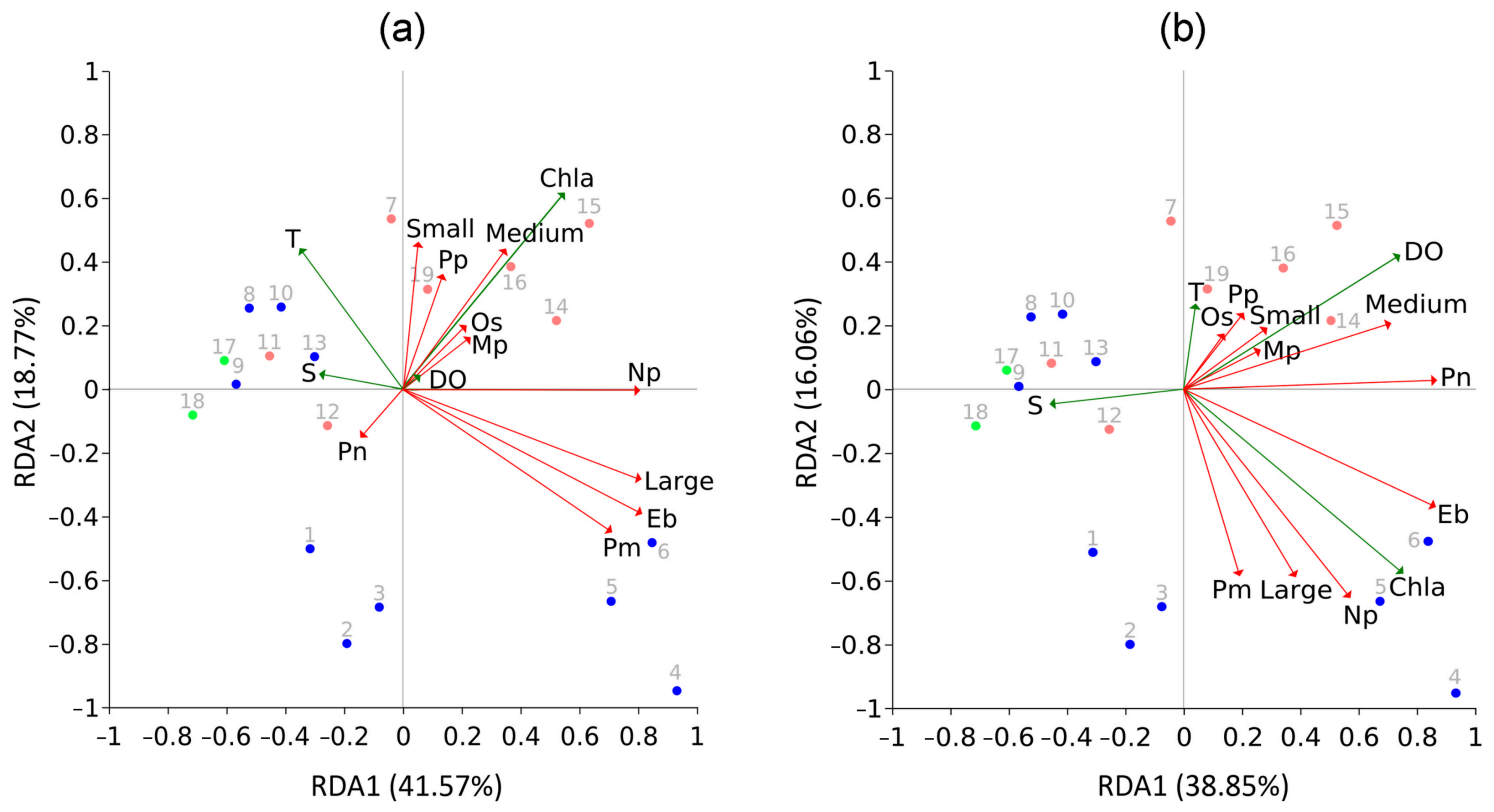
Ordination triplot of samples as inferred from redundancy analysis (RDA) based on log(x + 1)-transformed carbon biomass values (mg C m^−3^) for the major species and size classes in relation to the environmental variables (**a**) above the thermocline and (**b**) below the thermocline off the Kuril Islands. Abbreviations of the major copepod species (red arrows) are as follows: Np, *Neocalanus plumchrus*; Eb, *Eucalanus bungii*; Pp, *Paracalanus parvus*; Pm, *Pseudocalanus minutus*; Pn, *Pseudocalanus newmani*; Mp, *Metridia pacifica*; Os, *Oithona similis*. Small, body length from 0.5 to 1 mm; Medium, body length from 1 to 2 mm; Large, body length > 2 mm. Blue, peach, and green dots represent stations in the OCW, SWCW, and KEW, respectively. Abbreviations of the environmental variables (green arrows) are as follows: T, water temperature; S, salinity; DO, dissolved oxygen concentration; Chla, chlorophyll *a* concentration (see Section 2).

To assess the relationship between the copepod size structure and the environmental variables, a Redundancy Analysis (RDA) was performed. The first two canonical axes accounted for 60.34% of the explained variance, with RDA1 explaining 41.57% and RDA2 explaining 18.77% (*p* < 0.001) (Figure 7a). The RDA model based on the carbon biomass data for the layer above the thermocline explained 67.6% of the copepod variability (R^2^ = 0.61, F = 6.12, *p* < 0.001); chlorophyll *a* (38.2%, *p* < 0.001) and temperature (26.1%, *p* < 0.01) had the highest explanatory power. The water salinity (20.4%, *p* < 0.01) and dissolved oxygen (15.3%, *p* < 0.05) were not the strongest explanatory predictors, which suggested a secondary role. The first two canonical axes accounted for 54.91% of the explained variance, with RDA1 explaining 38.85% and RDA2 explaining 16.06% (*p* < 0.05) (Figure 7b). The RDA model based on the carbon biomass data for the layer below the thermocline explained 61.7% of the total variance (R^2^ = 0.55, F = 5.89, *p* < 0.001). Dissolved oxygen explained the largest proportion of variance (34.7%, *p* < 0.001), followed by chlorophyll *a* (28.4%, *p* < 0.01), salinity (22.1%, *p* < 0.01), and temperature (14.8%, *p* < 0.05).

Spearman’s correlation analysis demonstrated thermocline-specific differences in the relationships between copepod size structure, functional characteristics, and environmental variables (Table 5). In the layer above the thermocline, the carbon biomass of *Neocalanus plumchrus*, *Eucalanus bungii*, and *Pseudocalanus newmani* showed statistically significant negative correlations with temperature (*p* < 0.05), whereas the carbon biomass of *Paracalanus parvus* was significantly positively correlated with temperature. Below the thermocline, the carbon biomass of *Eucalanus bungii*, *Neocalanus plumchrus*, and *Pseudocalanus minutus* exhibited a significant negative correlation with salinity (*p* < 0.05). The diversity indexes showed statistically significant negative correlations with chlorophyll *a* concentration. The carbon biomass of small- and medium-sized classes was significantly positively correlated with temperature and dissolved oxygen concentration, respectively; the carbon biomass of the large-sized class demonstrated a negative correlation with temperature and salinity and a positive correlation with chlorophyll *a* concentration. Both the NBSS slope and NBSS R^2^ were significantly negatively correlated with temperature and salinity across all layers (*p* < 0.05) (Table 5). Low values of these environmental factors were related to NBSS slopes, indicating an enhanced energy transfer efficiency and dominance of the large-sized class of copepods in the OCW. The NBSS intercept had a significant relationship with high temperature and salinity above the thermocline (*p* < 0.05), with a peak in the KEW, and showed a tendency to decrease at higher latitudes. Considered together, the results of the RDA and Spearman’s correlation analysis demonstrated that chlorophyll *a* and temperature were the primary drivers of variations in the copepod size structure above the thermocline, while dissolved oxygen and chlorophyll *a* were the dominant predictors below the thermocline.

## 4. Discussion

The Kuril Islands, stretching over 1000 km from north to south, are characterized by high volcanic activity and a system of cold and warm currents. This combination of geological and oceanographic processes creates a highly dynamic environment with sharp gradients in temperature, salinity, and nutrient availability supporting a unique pelagic ecosystem [39,40,41]. In recent decades, studies have focused mainly on hydrodynamic and biological characteristics of the pelagic ecosystem in the Northwest Pacific, including advective processes [26,42,43], climatic regime shifts [17,25,26,44], sea surface temperature [45,46], chlorophyll *a* concentration [47,48], and fish abundance [8,24,49]. Although numerous studies have considered the subarctic pelagic ecosystem, the effect of environmental factors on the energy transfer from small to large organisms in such a fish-rich pelagic ecosystem as the waters along the Kuril Islands remains poorly understood. This ecosystem is among the world’s “biodiversity hotspots”, particularly rich in fisheries resources including walleye pollock, salmon, cod, mackerel, grenadier, sardine, crabs, sea urchins, prawn, saury, perch, squid, and seaweeds [6]. The results of the present study have confirmed the existence of three clearly distinguished water masses (OCW, SWCW, and KEW) along the Kuril Islands characterized by different environmental conditions. In recent years, the subarctic OCW has shown an increase in the mean temperature [5,50]. Considering that high temperatures may inhibit spawning in lipid-rich boreal copepods with 1–2-year life cycles [51,52], the relatively warm conditions could explain their relatively low abundance observed during our study. The area south of Urup Island (45°N) was influenced by relatively warm waters of the SWCW, a mixture of subtropical waters from the Tsushima Current and freshened waters from the Sea of Okhotsk. Thus, the water transported by the SWCW may be considered a suitable habitat for both boreal and subtropical copepods. The southern Kuril Islands area was influenced by warm waters of the KEW, where sea surface temperatures (SST) were higher than 17 °C, driven by the strong inflow of the Kuroshio Extension from the Pacific Ocean [12]. In this area, copepods were represented mostly by small-sized species, e.g., *Oithona* and *Oncaea*, which have multiple life cycles during the year and are abundant within the surface layer year-round. Thus, the temperature-driven water mass dynamics shape the quantitative distribution of copepods, which has important implications for ecosystem functioning and energy fluxes in the northwestern Pacific Ocean under current climate change scenarios.

In a previous study, we found that summer zooplankton assemblages comprised amphipods, appendicularians, copepods, chaetognaths, cladocerans, euphausiids, ostracods, salps, and pelagic larvae, with copepods being the most frequently occurring and abundant component [28]. Boreal copepods (e.g., *Eucalanus bungii*, *Neocalanus cristatus*, *Neocalanus plumchrus*, *Pseudocalanus newmani*, and *Pseudocalanus minutus*) tended to regions influenced by the subarctic OCW, which indicated their thermal preference for cold waters. This finding was not surprising, because these copepods are typical inhabitants of Northwest Pacific waters. Nevertheless, *Neocalanus plumchrus* in the earliest development stages was present in the SWCW and KEW, being transported there by the submerged Oyashio Current [23,53]. In contrast, subtropical copepods (e.g., *Clausocalanus arcuicornis*, *Paracalanus parvus*, and *Mesocalanus tenuicornis*) were indicators of the SWCW and KEW. Their occurrence as far north as Iturup Island during our study suggested increased northward transport of the SWCW to an area where this water had previously been absent [14]. The presence of cold-water species in the southern part of the study area and subtropical taxa as far north as Iturup Island suggested intensified water mixing and northward transport of warm waters. Overall, the copepod composition was tightly related to the water mass distribution, and the observed northward spread of subtropical species could serve as an early predictor of changing oceanographic conditions in the northwestern Pacific Ocean.

The spatial distribution of copepod carbon biomass varied considerably between the water masses off the Kuril Islands. Carbon biomass values did not differ between OCW and SWCW but were lower in KEW. The transport of nutrients from the Sea of Okhotsk through the straits [54] and intense physical processes can create favorable conditions for phytoplankton blooms in cold waters in the summer [55]. These blooms provided a food supply for large-sized copepods and resulted in higher carbon biomass compared to that in the KEW, which lacked such nutrient enrichment. Additionally, the highest carbon biomass was recorded above the thermocline, where it increased at higher latitudes and lower water temperatures but decreased with depth. Above the thermocline, the medium- and small-sized classes dominated in carbon biomass, whereas the large-sized class dominated below the thermocline, where the coldest temperatures favored cold-water copepods. Our results suggest that both horizontal (water mass) and vertical (thermocline) gradients shaped the copepod carbon biomass in the Kuril Islands pelagic ecosystem.

In terms of size class composition, the KEW differed from the SWCW and OCW. The warmest mean temperatures associated with the KEW likely promoted the growth of small-sized copepods, leading to the dominance of the <1 mm size class, which was consistent with the results by Wang et al. [56], who reported that copepod body size is inversely correlated with habitat temperature. The 1–2 mm size class was present at southern stations of the study region and was mainly influenced by the SWCW. This might be explained by the occurrence of large-sized copepods in the mixed water transported by the OCW. The environmental conditions of the OCW were distinct, characterized by the lowest temperatures. Therefore, the proportion of the >2 mm size class in the OCW was higher than in the other study areas, a pattern similar to that previously reported for cold waters off Japan [57].

In a balanced marine ecosystem, where energy is transferred without losses, the slope should be approximately −1.0. The slope is considered an index of productivity, transfer efficiency, and predation in the ecosystem, with steeper slopes indicating lower transfer efficiency and higher productivity and flatter slopes, vice versa, indicating higher transfer efficiency and lower productivity [22]. In the present study, the highest abundance of the small-sized class and the highest carbon biomass of the large-sized class shaped the size spectra of the copepod community in the pelagic ecosystem. The NASS slopes for each water mass were slightly higher than the theoretical −1, indicating a negative relationship between size intervals and abundance. The steepest NASS slopes were recorded for the OCW and KEW, suggesting a numerical dominance of small-sized copepods and a decline in their abundance with increasing body size. The flattest NASS slopes were observed in the SWCW, suggesting a more even distribution of copepod abundance across the size classes. The overall mean NASS slope values fit into the range optimal for marine ecosystems [22].

The NBSS slopes in the western North Pacific were typically negative [48,58,59], in contrast to those in the present study that were positive and showed an increase in carbon biomass with increasing copepod body size. It is important to note that the methodological choices, including the number and width of size classes and the use of length–carbon biomass regressions, might have affected the absolute values of NBSS slopes. Nevertheless, as evidenced by the consistent spatial patterns observed across the distinct water masses, each with contrasting environmental conditions, it was the ecological processes rather than methodological artifacts that were the primary causes of the differences in slope values. This was particularly evident in the oligotrophic waters (KEW), where the ecosystem functioned towards optimized utilization of bioresources and was characterized by a stable balance of carbon biomass across the size classes, which is typical of subtropical regions where nutrients and food sources are extremely scarce [34,35]. In contrast, the nutrient-rich waters (OCW) promoted phytoplankton blooms with elevated chlorophyll *a* concentrations, thereby increasing total carbon biomass through the growth of large-sized copepods. Our results are consistent with the findings of Tadokoro et al. [23], who reported that the biomass in cold waters may be accumulated in large-sized copepods. On the one hand, small-sized copepods dominated numerically, and, although they exhibited rapid reproduction and growth, their low energy transfer efficiency suggested limited consumption by animals of higher trophic levels. On the other hand, large-sized species were dominant in terms of biomass, actively accumulating nutrients during the short summer and transferring them to higher trophic levels with high efficiency. We assume that the positive NBSS slopes observed in the Kuril Islands region reflect a unique pattern of ecosystem functioning, indicative of efficient energy transfer mediated by large-sized copepods in the nutrient-rich northern waters (OCW). In contrast, the southern oligotrophic waters (KEW) exhibited a more balanced size-frequency distribution, likely reflecting adaptation to resource scarcity.

Environmental factors can explain the general variability in zooplankton assemblages, while their different combinations have different effects on size classes [19]. Our RDA and Spearman correlation analyses were largely corroborated by the identified significant relationships between environmental variables, thermocline depth, carbon biomass, and body lengths, which indicated the importance of environmental effects on the copepod size stratification along the Kuril Islands. We found that small-sized copepods were primarily limited by a combination of temperature and oxygen. In contrast, large-sized copepods were typically associated with areas of high primary production and deep-water layers, i.e., they were influenced by temperature and chlorophyll *a*.

Our study also revealed that environmental variables can cause significant vertical differences in copepod size structure between water masses. Above the thermocline, chlorophyll *a* and temperature turned out to be the main factors controlling the size distribution. This finding was consistent with previous studies showing that environmental factors were key regulators of vertical distribution of copepod biomass [59]. The strong negative correlation of the NBSS slope with temperature suggested that a positive slope, associated with efficient energy transfer, was dominant in the OCW, where copepods feed intensively during the summer season, accumulating lipid reserves. Below the thermocline, dissolved oxygen and chlorophyll *a* were the dominant predictors, reflecting a shift from thermal control to control limited by resources and oxygen. The positive correlation between large-sized copepods and chlorophyll *a* in deep layers is consistent with the hypothesis that sinking phytoplankton aggregates are consumed by deep-living diapausing copepods such as *Neocalanus plumchrus* and *Eucalanus bungii* [53,60]. In general, this finding confirms the importance of the thermocline as a critical ecological boundary: above the thermocline, temperature and food supply determine the accumulation of copepod carbon biomass; below the thermocline, oxygen concentration and sinking organic matter become the leading factors. This vertical partitioning may have direct influences on both the biological carbon pump and the availability of large-sized copepods as prey for organisms of higher trophic levels, including fish and whales.

It has been reported that variations in the size structure of marine zooplankton communities may be driven by either bottom-up or top-down processes [61,62]. The key factors determining the bottom-up control are the concentration of chlorophyll *a*, regulating the amount of phytoplankton, which is the main food supply for large-sized copepods; water temperature, which influences the metabolic rate, egg development, and life span of copepods (in warmer waters, development accelerates but body size is reduced); and dissolved oxygen, especially at great depths, which determines physiological condition, survivability, and avoidance of hypoxic zones [63]. The nutrient-rich OCW appeared to be dominated by a strong bottom-up control, supported by high phytoplankton abundance, which may have favored the dominance of large-sized copepods. In the oligotrophic KEW, a weak bottom-up control (low phytoplankton, high temperatures) was observed, possibly in combination with top-down pressure, which could explain the dominance of small-sized species. The transitional SWCW occupied an intermediate position, with moderate contributions from both types of control and a dominance of medium-sized copepods. These results suggested that bottom-up mechanisms could potentially explain the differences in copepod size spectra, as evidenced by the strong correlations of the NBSS slope with temperature and the high explained variance between chlorophyll *a* and temperature in the RDA models. An indirect indicator of top-down control in our study was the negative correlation between the Shannon–Wiener index and chlorophyll *a* concentration. Specifically, high food availability may have facilitated the dominance of a few competitively superior copepod species (e.g., *Neocalanus plumchrus*) that could suppress other species through exploitative competition or direct interference, ultimately reducing the overall community diversity. This pattern was consistent with competitive exclusion rather than resource limitation. Additionally, in the OCW, despite the strong correlations of large-sized species with temperature, the lack of correlations of the NASS parameters with environmental variables suggested that the predation pressure from pelagic fish may have influenced the abundance of large-sized copepods. However, it is important to emphasize that the presence of predators did not necessarily imply effective top-down regulation; effective regulation depended on whether consumption rates were sufficient to suppress growth of the prey population. In the nutrient-rich OCW, large-sized copepods apparently benefited from some physiological advantages (e.g., vertical migration, diapause, and size refuges) that enabled them to rapidly exploit phytoplankton blooms and maintain high population densities despite predation. In addition, predator avoidance strategies such as diel vertical migration, seasonal diapause, and size refuge effects may have further reduced the predation pressure on large-sized species. Collectively, these mechanisms could exclude the presence of predators from effective regulation. Therefore, we hypothesize that the copepod size distribution was related to adaptation to gradients of temperature, oxygen, and food supply, i.e., it was driven by bottom-up control, with predation potentially playing only a secondary, modulating role.

## 5. Conclusions

This is the first study focused on copepod size structure and energy flux (NASS/NBSS) in the fish-rich pelagic ecosystem of the Kuril Islands region. We have confirmed our hypothesis that water masses are the primary determinants of spatial and vertical variability in copepod size structure and energy flux in the Kuril Island ecosystem and that copepods can be used as an efficient indicator to assess the functioning of a pelagic ecosystem in relation to environmental conditions. The spatial and vertical variations in copepod abundance and carbon biomass revealed a clear north-to-south latitudinal gradient. The size structure distribution exhibited progressive downsizing from the northern Kuril Islands, where the cold OCW prevails, to the southern Kuril Islands, influenced by the warm SWCW and KEW. Three functional and relatively stable (ecologically defined and statistically supported) complexes, associated with the distinct water masses, were identified. The first complex was observed in the oligotrophic KEW, which had the highest temperature, salinity, and oxygen concentration values but the lowest chlorophyll *a* concentration. This complex was characterized by the lowest average carbon biomass, the dominance of the small-sized class (e.g., *Oithona similis*), a relatively low energy transfer efficiency, high productivity, and a less stable size structure. The second complex was identified in the SWCW (a transitional area), where temperature, salinity, and chlorophyll *a* concentration values were moderate, while oxygen concentration was the lowest. This complex was distinguished by the highest average carbon biomass, the dominance of the medium-sized class (e.g., *Metridia pacifica*, *Paracalanus parvus*, *Pseudocalanus minutus*, and *Pseudocalanus newmani*), an intermediate energy transfer efficiency, a moderate productivity, and a stable size structure. The third complex was observed in the OCW, which exhibited the lowest temperature and salinity, a moderate oxygen concentration, and the highest chlorophyll *a* concentration. This complex was characterized by the dominance of the large-sized class (e.g., *Eucalanus bungii* and *Neocalanus plumchrus*), a relatively high energy transfer efficiency, low productivity, and a stable size structure. Therefore, the pelagic ecosystem of the Kuril Islands comprised three functional complexes: KEW, which potentially contributed to overall resilience; SWCW, which acted as a dynamic transitional zone enhancing connectivity with adjacent waters; and OCW, which served as a core area of carbon biomass accumulation. Additionally, we found that some patterns were consistent between the layers above and below the thermocline, whereas other patterns were clearly opposite. Specifically, the latitudinal gradient in copepod size structure, the higher energy transfer efficiency in the OCW, and the distinction of three functional complexes were consistent between the two layers. In contrast, the dominant environmental predictors (chlorophyll *a* above the thermocline vs. dissolved oxygen below the thermocline), the species–environment relationships, and the vertical shift in carbon biomass peaks (in the SWCW above the thermocline vs. in the OCW below the thermocline) showed opposite patterns between the two layers. The findings for the KEW should be interpreted with caution because of the limited number of stations, and further studies with more extensive spatial sampling are needed to confirm the observed patterns. Our study highlights the usefulness of stratified sampling combined with functional approaches for understanding the complex dynamics in a fish-rich pelagic ecosystem. The findings also provide a scientific basis for modeling energy fluxes and practical approaches to fisheries management under ongoing climate warming and associated ecosystem shifts in the northwestern Pacific Ocean.

## Figures and Tables

**Table 1:** Environmental variables (mean ± SD), copepod average abundance (Abun), and average carbon biomass (CB) above (up) and below (lo) the thermocline at the stations influenced by the Oyashio Current Water (OCW), Soya Warm Current Water (SWCW), and Kuroshio Extension Water (KEW) during summer.

Variables	OCW	SWCW	KEW	One-Way ANOVA
T_up_	7.60 ± 3.94	14.65 ± 3.21	17.47 ± 0.19	*
T_lo_	2.81 ± 0.36	6.00 ± 1.25	3.44 ± 0.1	*
S_up_	33.04 ± 0.17	33.52 ± 0.29	33.95 ± 0.35	*
S_lo_	33.31 ± 0.06	33.46 ± 0.13	33.40 ± 0.29	*
Chla_up_	0.88 ± 0.34	0.98 ± 0.32	0.94 ± 0.28	*
Chla_lo_	0.41 ± 0.12	0.20 ± 0.04	0.20 ± 1.8	*
DO_up_	6.27 ± 0.53	5.98 ± 0.55	6.57 ± 0.42	*
DO_lo_	5.31 ± 0.55	5.18 ± 0.43	5.17 ± 0.34	*
Abun_up_	3642.5 ± 450.3	3396.9 ± 443.5	368.0 ± 61.9	*
Abun_lo_	2317.3 ± 251.6	1296.9.4 ± 130.8	297.2 ± 56.3	ns
CB_up_	192.2 ± 17.8	227.1 ± 21.4	26.8 ± 2.1	*
CB_lo_	113.9 ± 9.6	91.8 ± 6.9	20.9 ± 1.9	ns

Notes: Differences between groups were tested by one-way ANOVA. * *p* < 0.05; ns means that the difference is not significant.

**Table 2:** Indicator Value Index (IndVaI) of copepods from the Kuroshio Extension Water (KEW), the Soya Warm Current Water (SWCW), the Oyashio Current Water (OCW) and from both the Pacific (Pacific) and Sea of Okhotsk (Okhotsk) areas off the Kuril Islands. Significant *p*-values are highlighted in bold.

Species	Parameter	IndVaI, %	*p*-Value
*Calanus pacificus*	Pacific */–	75.1/–	**0.03**/–
*Clausocalanus arcuicornis*	SWCW */SWCW	61.5/41.9	**0.02**/0.06
*Copepoda nauplii*	KEW */–	72.2/–	**0.04**/–
*Eucalanus bungii*	–/OCW *	–/71.4	–/**0.02**
*Mesocalanus tenuicornis*	SWCW */SWCW	84.5/17.7	**0.0006**/0.64
*Metridia pacifica*	–/SWCW * and OCW	–/81.9 and 44.1	–/**0.01** and 0.17
*Neocalanus cristatus*	–/OCW *	–/75.2	–/**0.008**
*Neocalanus plumchrus*	OCW */OCW *	81.6/78.8	**0.02**/**0.03**
*Paracalanus parvus*	SWCW */–	87.5/–	**0.0001**/–
*Pseudocalanus minutus*	Pacific */OCW	100/67.8	**0.007**/**0.01**
*Pseudocalanus newmani*	OCW */Okhotsk *	100/77.2	**0.01**/**0.02**
*Oithona similis*	KEW */OCW	75.4/38.8	**0.02**/**0.004**

Notes: En-dash (–) means that the species was not an indicator. The Pacific area covers sampling stations 2–6, 8–10, and 12–19; the Sea of Okhotsk area covers sampling stations 1, 7, and 11. The values before the slash were obtained above the thermocline; after the slash, below the thermocline. The asterisk (*) indicates strong indicator taxa of water mass (index of more than 70%).

**Table 3:** Size classes and ranges of average/relative copepod abundance and carbon biomass within groups delineated by nMDS analysis.

Body Size Class	OCW	SWCW	KEW
Total abundance (ind. m^−3^/%)			
0.5–1 mm	2919.3/48.9	1591.0/33.9	327.3/49.2
1–2 mm	2453.4/41.1	2694.7/57.4	298.1/44.8
>2 mm	586.9/9.8	408.2/8.7	39.6/5.9
Abundance (ind. m^−3^/%) above the thermocline			
0.5–1 mm	1705.2/46.8	1079.5/31.8	212.5/61.2
1–2 mm	1589.8/43.6	2042.1/60.1	125.2/36.1
>2 mm	347.4/9.6	275.5/8.1	9.3/2.7
Abundance (ind. m^−3^/%) below the thermocline			
0.5–1 mm	1214.1/52.4	511.5/39.4	114.8/36.1
1–2 mm	863.5/37.2	652.6/50.3	172.9/54.4
>2 mm	239.5/10.4	132.7/10.3	30.3/9.5
Total carbon biomass (mg C m^−3^/%)			
0.5–1 mm	5.9/1.9	25.6/8.0	22.6/47.6
1–2 mm	132.0/43.2	199.3/62.5	14.6/30.7
>2 mm	167.6/54.8	94.0/29.4	10.3/21.7
Carbon biomass (mg C m^−3^/%) above the thermocline			
0.5–1 mm	3.5/1.8	17.7/7.8	14.5/58.7
1–2 mm	89.5/46.5	148.2/65.3	5.5/22.3
>2 mm	99.2/51.7	61.2/26.9	4.7/19.0
Carbon biomass (mg C m^−3^/%) below the thermocline			
0.5–1 mm	2.4/2.1	7.9/8.7	8.1/35.5
1–2 mm	42.5/37.5	51.1/55.5	9.1/39.9
>2 mm	68.4/60.4	32.8/35.8	5.6/24.5

**Table 4:** Normalized size spectra of copepods from the Kuroshio Extension Water (KEW), the Soya Warm Current Water (SWCW), and the Oyashio Current Water (OCW).

Water Mass	Slope (*a*)	Intercept (*b*)	R^2^	Probability Level
NASS				
OCW	–1.309, Very high	–1.313, Very high	0.708, Stable	0.00005 ***
SWCW	–1.026, High	–1.504, High	0.540, Moderate	0.0046 ***
KEW	–1.322, Very high	–1.350, Very high	0.615, Stable	0.0731 ns
NBSS				
OCW	2.231, Low	–3.940, Moderate	0.738, Stable	0.00001 ***
SWCW	1.167, Low	–3.067, Moderate	0.400, Moderate	0.0109 **
KEW	–0.052, Low	–1.725, High	0.295, Unstable	0.9086 ns

Notes: Differences between the areas were tested by the Mann–Whitney *U*-test. ** *p* < 0.05; *** *p* < 0.01; ns means that the difference is not significant. The slope (*a*) represents energy transfer efficiency, the intercept (*b*) indicates productivity, and the regression coefficient (R^2^) is a proxy to estimate assemblage stability.

**Table 5:** Results of Spearman’s rank-order correlation analysis showing relationships between the functional characteristics of copepods and the environmental variables off the Kuril Islands in the summer of 2024.

Copepods	Variable							
	T_up_	T_lo_	S_up_	S_lo_	DO_up_	DO_lo_	Chla_up_	Chla_lo_
CB	−0.30	0.02	−0.23	**−0.54**	−0.06	0.33	0.08	0.03
Ns	0.29	−0.10	0.24	0.07	0.25	−0.20	0.33	**−0.47**
Mi	0.37	−0.11	0.39	0.29	0.27	−0.36	0.19	**−0.45**
Pe	−0.10	−0.31	0.09	0.01	0.19	0.03	0.18	−0.38
Hi	0.06	−0.36	0.18	0.04	0.42	−0.10	0.31	**−0.53**
NASS_a_	−0.04	−0.06	0.11	−0.20	0.14	0.12	0.09	−0.30
NASS_b_	−0.36	−0.38	−0.27	−0.08	0.23	−0.29	0.03	0.31
NASSR_2_	−0.43	−0.37	−0.19	0.01	0.12	−0.42	−0.17	0.34
NBSS_a_	**−0.80**	**−0.49**	**−0.66**	**−0.46**	0.15	0.01	−0.01	0.16
NBSS_b_	**0.66**	0.29	**0.58**	0.30	0.02	−0.13	0.02	−0.15
NBSSR_2_	**−0.82**	**−0.66**	**−0.46**	−0.39	0.38	−0.18	−0.06	0.29
CB Np	−**0.70**	−0.45	**−0.46**	**−0.51**	0.22	0.02	0.07	0.12
CB Eb	−**0.70**	−0.44	−0.36	**−0.65**	0.26	0.04	−0.01	0.17
CB Pp	**0.75**	**0.71**	**0.61**	0.35	−0.37	0.39	0.14	−0.22
CB Pm	−0.44	−0.30	−0.36	**−0.58**	0.36	0.22	0.29	−0.01
CB Pn	−0.43	−0.38	**−0.61**	−0.08	0.07	−0.37	−0.23	0.16
CB Mp	0.29	−0.04	0.18	−0.11	0.19	−0.25	0.35	−0.41
CB Os	0.24	0.13	0.35	0.10	0.01	−0.04	−0.03	0.06
CB Small Size	**0.62**	0.10	0.37	0.10	0.03	−0.08	−0.04	0.05
CB Medium Size	0.06	0.32	−0.18	−0.33	−0.23	**0.51**	0.21	−0.14
CB Large Size	**−0.70**	−0.36	−0.39	**−0.58**	0.13	0.11	−0.01	**0.52**

Notes: CB, carbon biomass; Ns, number of species; Mi, Margalef’s index; Pe, Pielou’s evenness; Hi, Shannon–Wiener diversity index; NASS_a_, normalized abundance size spectra slope; NASS_b_, NASS intercept; NBSS_a_, normalized carbon biomass size spectra slope; NBSS_b_, NBSS intercept; NASSR_2_, R–Squared NASS; NBSSR_2_, R–Squared NBSS; Np, *Neocalanus plumchrus*; Eb, *Eucalanus bungii*; Pp, *Paracalanus parvus*; Pm, *Pseudocalanus minutus*; Pn, *Pseudocalanus newmani*; Mp, *Metridia pacifica*; Os, *Oithona similis*; Small Size, copepods with a body length from 0.5 to 1 mm; Medium Size, copepods with a body length from 1 to 2 mm; Large Size, copepods with a body length >2 mm; T, temperature; S, salinity; DO, dissolved oxygen concentration; Chla, chlorophyll *a* concentration; up, above the thermocline; lo, below the thermocline. Significant correlation coefficients (*p <* 0.05) are highlighted in bold.

## Data Availability

Data are contained within the article and the Supplementary Materials.

## Supplementary Materials

The following supporting information can be downloaded at https://www.mdpi.com/article/10.3390/biology15151293/s1. Table S1: Summary of sampling stations off the Kuril Islands during the summer of 2024. T—temperature (°C); S—salinity (PSU); DO—dissolved oxygen concentration (mL L^−1^); Chla—chlorophyll *a* concentration (µg L^−1^) above (Up) the thermocline and below (Lo) the thermocline; Abun—copepod abundance (ind. m^−3^); Biom—copepod biomass (mg WW m^−3^); CB—copepod carbon biomass (mg C m^−3^); KEW—Kuroshio Extension Water; SWCW—Soya Warm Current Water; OCW—Oyashio Current Water.
