# Supplementary material for: Effect of Environmental Conditions on Copepod Size Structure and Energy Flux in the Pelagic Ecosystem of the Kuril Islands Region
Source: Biology (Basel). 2026 Aug 4;15(15):1293. doi: 10.3390/biology15151293 (PMC13465924; doi:10.3390/biology15151293)
Supplement: Supplementary file 1 [file biology-15-01293-s001.zip › biology-4438010-supplementary.pdf]

Supplementary information

Table S1. Summary of sampling stations off the Kuril Islands during the summer of 2024. T—temperature (°C); S—salinity (PSU); DO—dissolved oxygen concentration (mL L^{-1}); Chla—chlorophyll *a* concentration (μg L^{-1}) above (Up) the thermocline and below (Lo) the thermocline; Abun—copepod abundance (ind. m^{-3}); Biom—copepod biomass (mg WW m^{-3}); CB—copepod carbon biomass (mg C m^{-3}); KEW—Kuroshio Extension Water; SWCW—Soya Warm Current Water; OCW—Oyashio Current Water.

Stn.	Date	Layer	Water mass	T	S	DO	Chla	Abun	Biom	CB
1	17.08.2024	Up	OCW	3.9	33.0	6.8	0.9	2690.4	976.3	62.4
1	17.08.2024	Lo	OCW	2.3	33.3	5.4	0.4	2991.2	1334.6	85.4
2	19.08.2024	Up	OCW	2.9	33.3	5.2	0.4	1227.4	1006.4	64.4
2	19.08.2024	Lo	OCW	2.7	33.4	4.7	0.5	3002.7	1785.5	114.2
3	20.08.2024	Up	OCW	3.1	33.2	6.0	0.3	2941.7	1597.6	102.2
3	20.08.2024	Lo	OCW	2.7	33.3	5.4	0.5	2746.4	1899.8	121.5
4	20.08.2024	Up	OCW	7.6	33.0	6.5	0.7	8024.5	5036.5	322.3
4	20.08.2024	Lo	OCW	2.7	33.4	4.8	0.4	7016.4	4415.6	282.6
5	22.08.2024	Up	OCW	6.4	32.9	6.5	1.4	6339.2	8290.1	530.5
5	22.08.2024	Lo	OCW	2.5	33.3	6.6	0.2	1995.1	1884.0	120.5
6	23.08.2024	Up	OCW	4.6	33.1	6.3	0.8	4900.1	7298.5	467.1
6	23.08.2024	Lo	OCW	3.4	33.2	5.7	0.3	2788.5	4028.4	257.8
7	27.08.2024	Up	SWCW	16.3	33.2	5.8	1.2	4674.1	3584.1	229.3
7	27.08.2024	Lo	SWCW	5.3	33.4	5.8	0.2	1994.3	1059.0	67.7
8	29.08.2024	Up	OCW	11.4	33.2	5.9	1.0	3720.9	1323.1	84.6
8	29.08.2024	Lo	OCW	3.2	33.3	5.3	0.3	513.8	391.7	25.1
9	29.08.2024	Up	OCW	11.1	33.2	5.9	1.0	2291.7	828.6	53.1
9	29.08.2024	Lo	OCW	3.2	33.3	5.3	0.3	604.1	475.7	30.4
10	29.08.2024	Up	OCW	11.3	32.9	6.8	1.3	2066.7	1741.3	111.4
10	29.08.2024	Lo	OCW	2.5	33.3	5.3	0.2	694.5	586.1	37.5
11	02.09.2024	Up	SWCW	19.2	33.4	5.4	0.6	1950.8	1360.4	87.1
11	02.09.2024	Lo	SWCW	7.4	33.6	5.9	0.2	959.5	629.7	40.3
12	04.09.2024	Up	SWCW	9.4	33.9	6.7	0.8	1590.4	1783.0	114.1
12	04.09.2024	Lo	OCW	2.1	33.4	4.9	0.1	875.7	1152.2	73.7
13	04.09.2024	Up	OCW	13.3	32.8	6.8	1.0	2222.8	1940.4	124.1
13	04.09.2024	Lo	OCW	2.5	33.4	4.6	0.1	819.5	909.5	58.2
14	05.09.2024	Up	SWCW	11.9	33.2	6.2	1.5	3863.1	3893.6	249.2
14	05.09.2024	Lo	OCW	3.5	33.3	5.6	0.2	1329.6	2437.0	155.9
15	09.09.2024	Up	SWCW	16.8	33.4	5.8	1.0	4684.7	5695.6	364.5
15	09.09.2024	Lo	OCW	8.1	33.4	5.5	0.3	1399.8	2100	136.1
16	09.09.2024	Up	SWCW	12.9	33.9	6.6	1.2	3705.1	4799.5	307.1
16	09.09.2024	Lo	OCW	2.7	33.3	5.6	0.1	1467.9	1883.9	120.5
17	09.09.2024	Up	KEW	16.3	33.7	6.9	1.0	680.9	752.8	48.1
17	09.09.2024	Lo	OCW	2.9	33.4	5.4	0.2	346.9	361.2	23.1
18	09.09.2024	Up	KEW	17.6	34.2	6.3	0.8	55.1	85.6	5.4
18	09.09.2024	Lo	OCW	3.9	33.4	5.0	0.2	247.5	294.0	18.8
19	10.09.2024	Up	SWCW	18.8	33.6	5.3	0.6	3309.7	3723.3	238.2
19	10.09.2024	Lo	OCW	5.2	33.4	5.1	0.2	1154.5	1449.7	92.7
